# Relationship between platelet count and severity of neonatal respiratory distress syndrome

**DOI:** 10.1186/s13052-024-01762-2

**Published:** 2024-10-08

**Authors:** Ying Zeng, Hai ying Yi, Yuan He, Bin Gan, Xian Wei, Jie Huang, Shu jie Yang

**Affiliations:** Department of Neonatology, Xiaogan Hospital Affiliated to Wuhan University of Science and Technology, 6 Plaza Road, Xiaogan, Hubei 432003 China

**Keywords:** NRDS, Platelet, Severity, Risk factor

## Abstract

**Background:**

Neonatal respiratory distress syndrome (NRDS) is a primary cause of morbidity and mortality in premature infants. Platelets have a unique role in lung repair and remodeling. This study aimed to determine the relationship between platelet count and NRDS severity.

**Methods:**

The study included 234 newborns diagnosed with NRDS from January 2019 to August 2023. This study employed two methods of grouping: the first based on platelet count, dividing participants into thrombocytopenia (platelet count < 150 × 10^9^/L, *n* = 50) and non-thrombocytopenia groups (platelet count ≥ 150 × 10^9^/L, *n* = 184), and the second based on the severity of NRDS, categorizing them into severe (*n* = 24) and mild-moderate (*n* = 210) groups. Within the first grouping method, the thrombocytopenia group was further subdivided into moderate-severe group (platelet count < 100 × 10^9^/L, *n* = 4) and mild group (platelet count was between 100.0 × 10^9^/L and 150.0 × 10^9^/L, *n* = 46). This study aimed to analyze the clinical characteristics of NRDS with thrombocytopenia, explore the correlation between platelet count and clinical indicators of NRDS. Binary Logistic regression analysis was employed to identify independent risk factors for thrombocytopenia in NRDS.

**Results:**

A higher proportion of newborns in the severe group exhibited thrombocytopenia (severe group = 41.7%, mild-moderate group = 19.0%). Hospital stay, ventilation time, oxygen therapy duration were longer in the thrombocytopenia group compared to the non-thrombocytopenia group. Hospital stay, ventilation time, oxygen therapy duration, chest radiography score, and C-reactive protein (CRP) levels were inversely associated with platelet count. Conversely, Apgar scores at 1 and 5 min, gestational age, and birth weight showed positive correlations with platelet count. Point-biserail correlation showed that thrombocytopenia was more likely to occur in newborns whose mothers had gestational hypertension, and the lower platelet count, the more severe NRDS. Oxygen therapy duration, birth weight < 1500 g, gestational hypertension and CRP levels emerged as independent risk factors for thrombocytopenia in NRDS. All differences were statistically significant (*p* all < 0.05).

**Conclusion:**

NRDS accompanied by thrombocytopenia indicates a more severe condition and poorer clinical outcomes. It is hypothesized that NRDS with thrombocytopenia involves a complex multifactorial etiology, including severe lung inflammation.

**Supplementary Information:**

The online version contains supplementary material available at 10.1186/s13052-024-01762-2.

## Background

Neonatal respiratory distress syndrome (NRDS) is a common respiratory condition in newborns, primarily resulting from pulmonary surfactant (PS) deficiency [1], which is prevalent among preterm and/or low birth weight neonates [2]. It is the main cause of morbidity and death in premature babies, with mortality rate in low income countries at times close to 100% [3]. Therefore, it is necessary to establish a biochemical marker to predict the severity of NRDS, also with the aim of implementing strategies oriented to optimize the management of patients, and minimizing the adverse outcomes.

Platelets participate in the pathogenesis of NRDS through the coagulation pathway, particularly in cases of severe NRDS [4]. Approximately 1–4% of newborns experience thrombocytopenia at birth, with rates in Neonatal Intensive Care Units (NICU) reaching 10–25%, and up to 50% in newborns with a birth weight under 1000 g [5]. The relationship between platelet count and acute respiratory distress syndrome in adults has been extensively studied, but data on neonates are lacking. This study aims to analyze the clinical characteristics of NRDS with thrombocytopenia, explore the relationship between reduced platelet count and NRDS severity, investigate the diagnostic capability of C-reactive protein (CRP), birth weight < 1500 g, oxygen therapy duration, and gestational hypertension for reduced platelet counts, and offer novel insights for the clinical management of NRDS.

## Methods

### Participants

This retrospective study involved 234 newborns diagnosed with NRDS within 24 h after birth at the NICU of Xiaogan Hospital Affiliated to Wuhan University of Science and Technology, in China, from January 2019 to August 2023. Clinical data were gathered from the electronic medical record system. According to the study of Giedion et al. [6]. the changes of chest radiographs on admission were divided into IV grades. I: Decreased translucency, uniformly scattered fine granular and lattice shadows were seen in both lungs. II: On the basis of I, the transmittance of the two lungs further decreased, and the air bronchial sign was seen. III: On the basis of II, the translucency of two lungs decreased even more, and the heart and diaphragm contour were blurred. IV: Complete involvement of both lungs, even with white lungs. Grade I recorded 1 point, and so on, the chest radiograph findings with a score 1 to 4.

### Inclusion criteria

1.The diagnosis of NRDS depends on progressive exacerbation of clinical signs and symptoms (tachypnea, nasal flaring, intercostal or subcostal or subxiphoid or suprasternal retractions, grunting, and cyanosis), arterial blood gas analysis (hypoxemia, elevated arterial CO_2_ and acidosis) and the chest radiographs fndings (grid and granular shadow, air bronchogram or atelectasis, and severe cases even present with white lung ) [2]. 2. Admission to NICU within 24 h after birth. 3. Chest radiography examination completed within 12 h of hospitalization, and with findings compatible with the disease and according to any of the 4 degrees of Giedion et al. [6]. If PS administration was necessary, the chest radiography should be performed prior to PS administration. 4. Patient data were complete, with no missing information.

### Exclusion criteria

1.Major congenital malformations. 2. Perinatal asphyxia complications, including hypoxic-ischemic encephalopathy, acute tubular necrosis, transient myocardial ischemia. 3. Combined with.

hematologic disorders, such as polycythemia, diffuse intravascular coagulation, aplastic anemia, thrombocytopenic purpura, leucocythemia, myelodysplastic syndromes. 4. Death within one week of birth. 5. Pregnant women taking medications that cause a decrease in platelet count. 6.Pregnant women with decreased platelet count, anemia, chorioamnionitis, immune diseases. 7.Positive blood culture. 8.Comorbidities or complications like necrotizing enterocolitis, meconium aspiration syndrome, hereditary metabolic disease.

### Groups classification

The study employed two methods of grouping: the first based on platelet count, and the second based on the severity of NRDS. The lowest platelet count in infants occurred by day 4 after birth [7], and such early thrombocytopenia accounts for 75% of cases in newborns admitted to NICU [8]. In this study, 234 participants were categorized into thrombocytopenia (platelet count < 150 × 10^9^/L, *n* = 50) and non-thrombocytopenia groups (platelet count ≥ 150 × 10^9^/L, *n* = 184) based on the lowest.

platelet count observed within 3–4 days after birth. The thrombocytopenia group (*n* = 50) was further divided into moderate-severe group (platelet count < 100 × 10^9^/L, *n* = 4) and mild group (platelet count was between 100.0 × 10^9^/L and 150.0 × 10^9^/L, *n* = 46). Following the classification by Brus et al. [9], the severity of NRDS was divided into severe and mild-moderate. Severe NRDS was defined as an oxygen requirement of more than 30% for adequate oxygenation, artificial ventilation dependency because of respiratory failure, and chest radiograph consistent with a score 3 or 4. Mild-to-moderate NRDS was defined as an extra oxygen requirement for adequate oxygenation, artificial ventilation or continuous positive airway pressure dependency because of respiratory failure, and chest radiograph consistent with a score 1 or 2. Accordingly, this study segmented 234 patients into severe group (*n* = 24) and mild-moderate group (*n* = 210).

### Statistical analysis

Data were analyzed using SPSS 26.0 software. Non-normally distributed data were presented as median and analyzed using the Mann-Whitney U test. Categorical data were reported as percentages and compared using the chi-squared test. Logistic binary regression was applied to identify risk factors for thrombocytopenia in NRDS. The association between platelet count and various parameters was evaluated using Spearman’s correlation coefficient. The correlation between platelet and gestational hypertension and severe NRDS were analyzed by point-biserail correlation.The receiver operating characteristic curve (ROC) was utilized to assess the predictive value of variables for identifying thrombocytopenia in NRDS. A *p* value < 0.05 was considered statistically significant. Graphs were generated with GraphPad Prism 8 software.

## Results

### Baseline characteristics

There were no significant differences in gender, cesarean section, twinning, gestational diabetes and hypothyroidism between the two groups (*p* > 0.05). There were significant differences in gestational age (weeks), birth weight (g), 1 min and 5 min Apgar scores, low Apgar score (1–5 min Apgar scores ≤ 7 points), and gestational hypertension between the two groups (*p* < 0.05). In the thrombocytopenia group, mean gestational age, birth weight and Apgar score at 1 and 5 min were lower; pregnant women were more likely to have hypertension, and low Apgar score occurred in more newborns (Table 1).

**Table 1:** Comparison of baseline conditions between the two groups

Characteristics	Thrombocytopenia group (*n* = 50)	Non-thrombocytopenia group (*n* = 184)	*x*^2^/Z	*P*-value
Gender (male)	33.0 (66.0%)	108.0 (58.7%)	0.876	0.349
Cesarean section	48.0 (96.0%)	170.0 (92.4%)	0.337	0.562
Twins	9.0 (18.0%)	45.0 (24.5%)	0.923	0.337
Gestational age (weeks)	33.3 (31.6, 36.1)	35.4 (34.0, 36.3)	-3.265	0.001**
Birth weight (g)	1845.0 (1407.5, 2500.0)	2400.0 (2022.5, 2827.5)	-3.827	<0.001***
Apgar 1 (1 min)	7.0 (5.0, 8.0)	8.0 (7.0, 8.0)	-3.887	<0.001***
Apgar 5 (5 min)	8.0 (8.0, 9.0)	9.0 (9.0, 10.0)	-4.788	<0.001***
Low Apgar score	32.0 (64.0%)	84.0 (45.7%)	5.295	0.021*
Pregnant mother (pregnancy combined)				
Hypothyroidism	6.0 (12.0%)	28.0 (15.2%)	0.328	0.567
Diabetes	16.0 (32.0%)	59.0 (32.1%)	0.000	0.993
Hypertension	14.0 (28.0%)	29.0 (15.8%)	3.926	0.048*

Note * *P*<0.05, ** *P*<0.01, *** *P*<0.001

### Clinical features

The hospital stay in the thrombocytopenia group was 13.5 days longer than in the non-thrombocytopenia group (*p* < 0.001). The ventilation time was 18 h longer in the thrombocytopenia group compared to the non-thrombocytopenia group (*p* < 0.05). The invasive ventilation time was 27.5 times longer in the patients belonging to the former bracket, though this difference was not statistically significant (*p* > 0.05). The non-invasive ventilation time was also longer (7.5 times more) in the patients belonging to the thrombocytopenia group (*p* < 0.05). Oxygen therapy duration, as well, was 144 hours longer in the same subjects (*p* < 0.001).   (remove the sentence "The thrombocytopenia group had more PS administration", as it is repeated twice). The thrombocytopenia group had more PS administrations (*p* < 0.05). The thrombocytopenia group was further divided into moderate-severe group and mild group. The hospital stay in the moderate - severe group was 27.0 days longer than in the mild group (*p* < 0.05). Oxygen therapy duration was 521 h longer in the moderate - severe group (*p* < 0.05) (Tables 2 and 3).

**Table 2:** Comparison of clinical features between the two groups

Item	Thrombocytopenia group (*n* = 50)	Non-thrombocytopenia group (*n* = 184)	*x*^2^/Z	*P*-value
Hospital stay (d)	24.5 (12.0, 43.3)	11.0 (9.0, 18.8)	-4.570	<0.001***
Pulse (bpm)	152.0 (145.0, 158.0)	152.5 (150.0, 156.0)	-0.497	0.619
Respiratory rate (bpm)	62.0 (60.0, 65.0)	62.0 (59.3, 65.0)	-0.719	0.472
Ventilation time (h)	88.0 (52.5, 202.3)	70.0 (47.0, 108.5)	-2.217	0.027*
Invasive ventilation time (h)	38.0 (0.0, 82.5)	10.5 (0.0, 47.8)	-1.851	0.064
Non-invasive ventilation time (h)	54.5 (39.8, 103.8)	47.0 (32.3, 71.0)	-1.969	0.049*
Oxygen therapy duration (h)	301.0 (156.3, 628.3)	157.0 (107.3, 259.0)	-4.005	<0.001***
PS administrations				
0–1 times	39.0 (78.0%)	165.0 (89.7%)	4.794	0.029*
2–3 times	11.0 (22.0%)	19.0 (10.3%)		

Note * *P*<0.05, *** *P*<0.001

**Table 3:** Further comparison of clinical features in thrombocytopenia group

Item	Mild (*n* = 46)	Moderate - severe (*n* = 4)	*x*^2^/Z	*P*-value
Hospital stay (d)	21.5 (11.5, 35.3)	48.5 (47.3, 60.3)	-2.469	0.014*
Pulse (bpm)	152.0 (145.8, 158.0)	150.0 (144.3, 155.8)	-0.681	0.496
Respiratory rate (bpm)	63.0 (60.0, 65.0)	59.5 (25.8, 61.8)	-1.875	0.061
Ventilation time (h)	88.0 (51.3, 202.3)	151 (69.8, 687.5)	-0.966	0.334
Invasive ventilation time (h)	38.0 (0.0, 82.5)	23.0 (0.0, 389.5)	-0.056	0.956
Non-invasive ventilation time(h)	53.0 (38.5, 84.5)	128 (61.8,306.0)	-1.789	0.074
Oxygen therapy duration (h)	279.5 (116.3, 574.5)	800.5 (426.3, 1174.8)	-2.038	0.042*
PS administrations				
0–1 times	37.0 (80.4%)	2.0 (50.0%)	3.588	0.250
2–3 times	9.0 (19.6%)	2.0 (50.0%)		

Note * *P*<0.05

### Complete blood count and chest radiography

The thrombocytopenia group had more newborns with chest radiography scores of 3–4 points (*p* < 0.01). The levels of white blood cell (WBC) count, hemoglobin (HB), and platelet crit (PCT) were lower in the thrombocytopenia group than in the non-thrombocytopenia group (*p* < 0.01). Conversely, CRP levels, platelet distribution width (PDW), and mean platelet volume (MPV) were higher in the thrombocytopenia group than in the non-thrombocytopenia group (*p* < 0.001) (Table 4).

**Table 4:** Comparison of complete blood counts and chest radiography in the two groups

Item	Thrombocytopenia group (*n* = 50)	Non-thrombocytopenia group (*n* = 184)	*x*^2^/Z	*P*-value
Chest radiography score			6.818	0.009**
1–2 points	39.0 (78.0%)	168.0 (91.3%)		
3–4 points	11.0 (22.0%)	16.0 (8.7%)		
WBC (×10^9/L)	7.8 (5.6, 11.7)	10.9 (8.5, 14.2)	-3.878	<0.001***
HB (g/L)	156.5 (134.5, 171.3)	166.0 (153.0, 179.8)	-2.904	0.004**
PDW (%)	16.9 (16.5, 17.3)	16.3 (16.1, 16.5)	-6.681	<0.001***
MPV (fL)	10.3 (9.7, 11.1)	9.3 (9.0, 9.7)	-7.372	<0.001***
PCT (%)	0.15 (0.13, 0.16)	0.26 (0.22, 0.28)	-10.252	<0.001***
CRP (mg/L)	1.9 (0.5, 10.4)	0.5 (0.3, 0.5)	-4.781	<0.001***

Note ** *P*<0.01, *** *P*<0.001

### Correlation analysis between platelet count and clinical indicators in the whole study sample (*n* = 234)

**Fig. 1:**
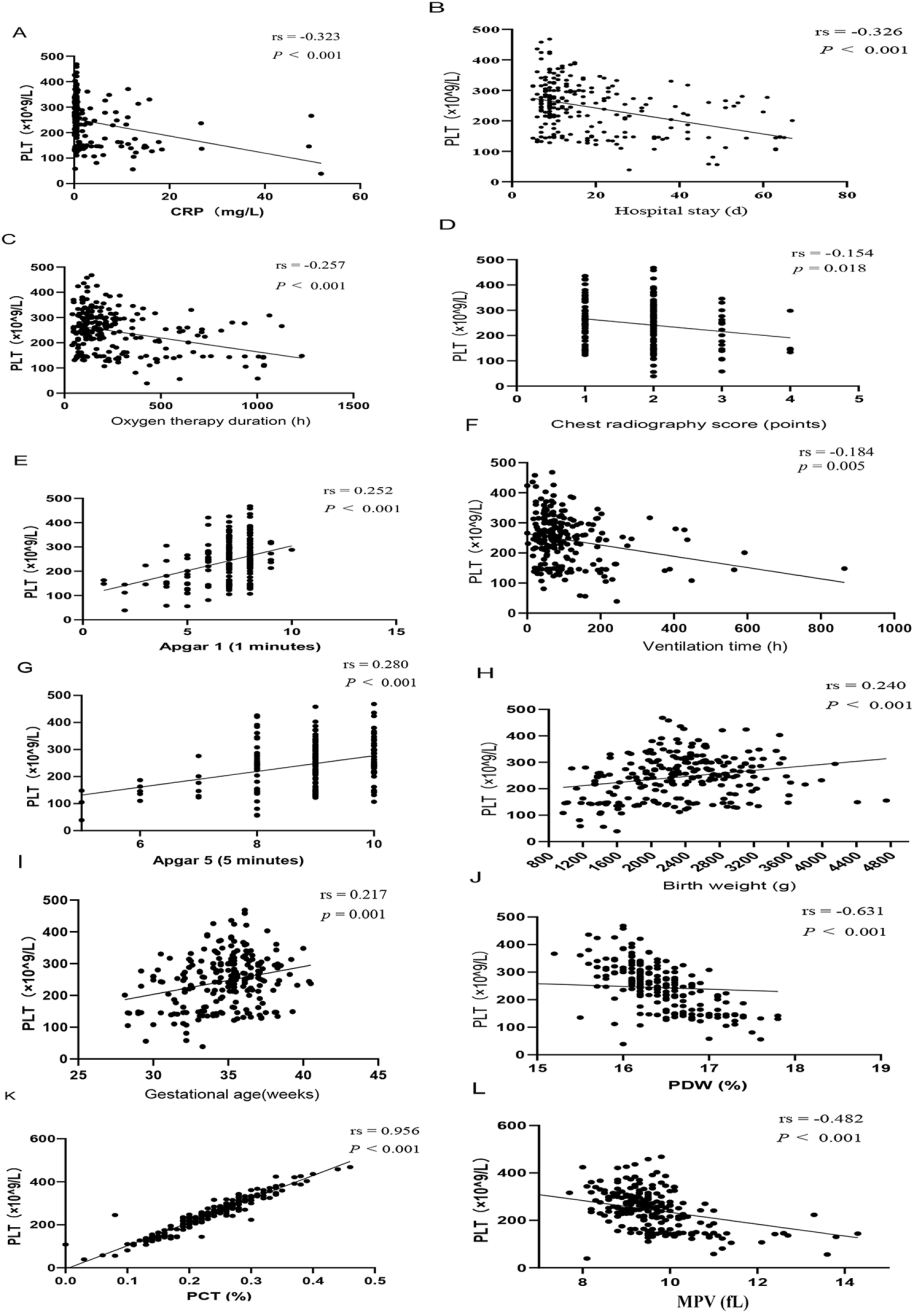
Correlation analysis of hospital stay, ventilation time, oxygen therapy duration, chest radiography score, CRP, PDW, MPV, Apgar 1 (1 min), Apgar 5 (5 min), gestational age, birth weight, PCT and platelet count in the entire study sample

The study found negative correlations between platelet count and hospital stay, ventilation time, oxygen therapy duration, chest radiography score, CRP, PDW, MPV (rs = -0.326, -0.184, -0.257, -0.154, -0.323, -0.631, -0.482, *p*<0.001, = 0.005,<0.001, = 0.018,<0.001,<0.001,<0.001, respectively). Conversely, positive correlations were observed between platelet count and Apgar scores at 1–5 min, gestational age, birth weight, and PCT (rs = 0.252, 0.280, 0.217, 0.240, 0.956, *p*<0.001,<0.001, = 0.001,<0.001,<0.001, respectively). In NRDS, the occurrence of thrombocytopenia was associated with longer ventilation time, oxygen therapy duration and hospital stay. Those with thrombocytopenia exhibited higher PDW, MPV, CRP levels and chest radiography score, and lower PCT, birth weight, 1-minute and 5-minute Apgar scores, and were younger in gestational age (Fig. 1). Point-biserail correlation showed that thrombocytopenia was more likely to occur in newborns whose mothers were affected by gestational hypertension (correlation coefficient was − 0.155, *p* = 0.018).

### Platelet count and disease severity

Patients were categorized into severe (*n* = 24) and mild-moderate (*n* = 210) groups based on NRDS severity. A lower platelet count was observed in the severe group( *p*<0.05) (Table 5). Point-biserail correlation showed that the lower platelet count, the more severe NRDS (correlation coefficient was − 0.192, *p* = 0.003).

**Table 5:** Platelet count and disease severity

Item	Severe group (*n* = 24)	Mild-moderate group (*n* = 210)	*x*^2^/Z	*P*-value
Thrombocytopenia (case)	10.0 (41.7%)	40.0 (19.0%)	6.599	0.01*
Non-thrombocytopenia (case)	14.0 (58.3%)	170.0 (81.0%)		
PLT (×10^9/L)	189.0 (145.0, 258.3)	255.0 (190.0, 300.3)	-2.632	0.008**

Note * *P*<0.05, ** *P*<0.01, *** *P*<0.001

### Analysis of risk factors for decreased platelet count in NRDS

**Fig. 2:**
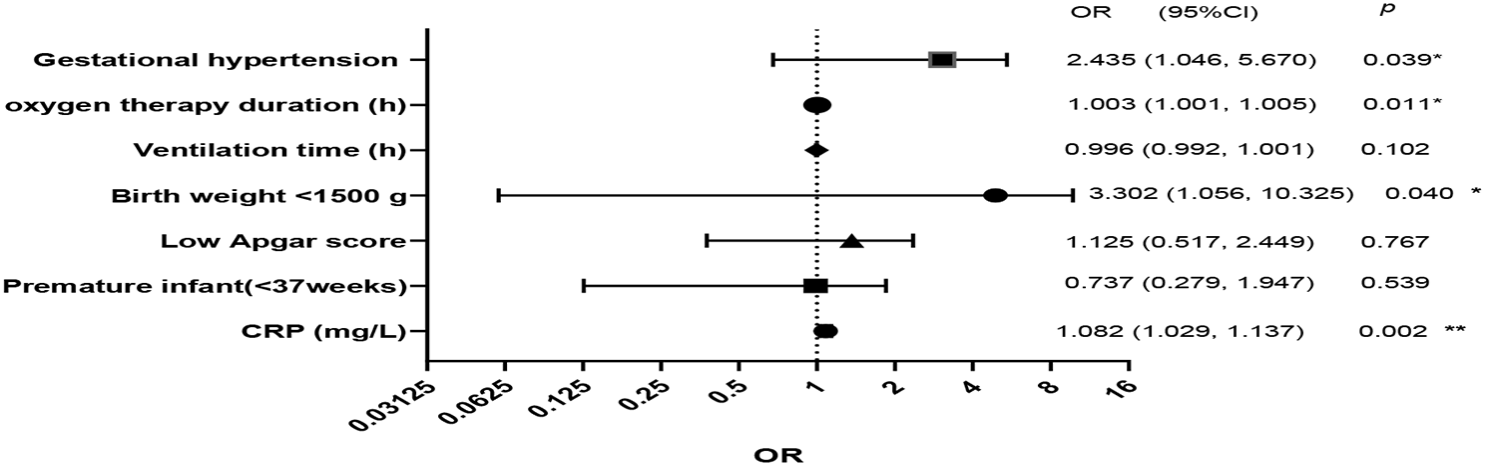
Binary Logstic regression analysis. * *P*<0.05, ** *P*<0.01

With thrombocytopenia as the dependent variable, clinical indicators such as gestational hypertension, oxygen therapy duration, ventilation time, birth weight < 1500 g, low Apgar score, prematurity (gestational age < 37 weeks) and CRP were analyzed through binary logistic regression. The analysis identified oxygen therapy duration (OR = 1.003, 95% CI = 1.001, 1.005), birth weight < 1500 g (OR = 3.302, 95% CI = 1.056, 10.325), gestational hypertension (OR = 2.435, 95% CI = 1.046, 5.670) and CRP levels (OR = 1.082, 95% CI = 1.029, 1.137) as independent risk factors for thrombocytopenia in NRDS (Fig. 2).

### ROC analysis

**Fig. 3:**
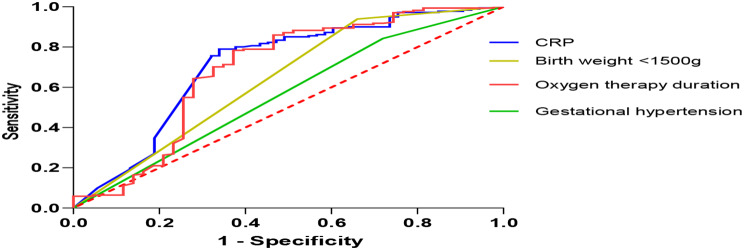
ROC analysis of CRP, birth weight < 1500 g, oxygen therapy duration, and gestational hypertension for the predictive effect of thrombocytopenia in NRDS

Further analysis of the ability of CRP, birth weight < 1500 g, oxygen therapy duration, and gestational hypertension in the predictive effect of thrombocytopenia in NRDS was performed. The area under curve (AUC) values, critical values, sensitivity and specificity of the relevant indices are demonstrated in Fig. 3; Table 6.

**Table 6:** Relevant data of ROC curve

Item	AUC	Cut-off point	Sensitivity (%)	Specificity (%)
CRP (mg/L)	0.716	0.730	68.0	78.8
Birth weight < 1500 g	0.637	0.500	34.0	93.5
Oxygen therapy duration (h)	0.685	508.0	32.0	90.2
Gestational hypertension	0.561	-1.0	1.0	0.0

## Discussion

Neonatal respiratory distress syndrome (NRDS) is a prevalent respiratory ailment in newborns. Existing studies suggest a strong correlation between NRDS severity and decreased platelet count, with a low platelet count serving as a biomarker for disease severity [10]. In our study, a greater number of newborns in the severe group developed thrombocytopenia. In NRDS, the occurrence of thrombocytopenia was associated with longer ventilation time, oxygen therapy duration and hospital stay, higher CRP levels and lower white blood cells, worst chest radiography severity scores (3–4) and more PS administrations. Overall, our study showed that thrombocytopenia in NRDS was associated with poorer clinical outcomes. The lung is considered a primary site for platelet production [11]. Platelets affect the activity of pulmonary vessels and play roles in regulating pulmonary vascular permeability and maintaining the integrity of the alveolar-capillary barrier, which are crucial for pulmonary defense and integrity [12]. Studies have found that severe NRDS is associated with increased coagulation, reduced fibrinolysis, and fibrin deposition in the lungs [13]. Coagulation factors leak into the alveolar space through damaged alveolar capillaries, and activated pro-coagulation factors and insufficient fibrinolysis lead to fibrin deposition and hyaline membrane formation in severe NRDS [14–16]. This process also promotes the aggregation of platelets and neutrophils, leading to increased platelet consumption [4]. Brus et al. [17] also noted a significant decline in platelet counts in severe NRDS, indicating activation of coagulation. In addition, studies have found that beyond primary surfactant deficiency, NRDS involves a harmful inflammatory response within the immature lungs [18, 19].The interaction between platelets and infection or immune-related cells can lead to increased platelet clearance and apoptosis [5]. Overall, NRDS may be an inflammatory disease, especially when accompanied by thrombocytopenia. It is speculated that severe NRDS may cause improper activity of white blood cells and platelets, as well as uncontrolled activation of clotting pathway. These factors may contribute to lung and vascular endothelium damage. Additionally, high-pressure and high-oxygen ventilation can cause oxidative stress injury to tissues, potentially promoting this activation process. We believe that the etiology of NRDS is complex and multifactorial, with various secondary factors exacerbating the inflammatory response in immature lungs. The relationship between platelets and NRDS is extremely complex, and platelets are not the only damaged target cells, but also accelerate the process of NRDS. These hypotheses may provide new insights into the pathophysiology of NRDS and warrant further study.

Our findings indicate that newborns with NRDS who developed reduced platelet counts were younger in gestational age, and lower in birth weight. It was possibly due to the immaturity of organs in infants and their limited capacity to compensate platelets consumption by increasing their production. Levy-Shraga et al. [20] found that women with gestational hypertension were more likely to exhibit decreased platelet counts. This study corroborated these findings, with a higher incidence of gestational hypertension observed in the thrombocytopenia group. The primary pathogenesis of gestational hypertension involves placental dysplasia and ischemia, leading to the release of oxidative stress factors, endothelial cell damage, platelet aggregation, and subsequently, platelet and systemic organ dysfunction [21]. Theoretically, various oxidative stress factors may enter the fetal circulation, potentially causing a range of adverse effects on the fetus/newborn. Although the precise pathogenesis of neonatal thrombocytopenia triggered by gestational hypertension remains unclear, it is widely accepted that hypertension contributes to fetal hypoxia, which in turn inhibits fetal megakaryocyte and platelet production [22]. Brus et al. [9] highlighted that, from the disease’s second day, platelet counts in severe NRDS cases were significantly lower than those in mild and moderate cases, and a lower 5-minute Apgar score was associated with reduced platelet counts. In this study, 1-minute and 5-minute Apgar scores in the thrombocytopenia with NRDS group were lower, showing positive correlation with platelet counts (rs = 0.252, 0.280, *p* all < 0.001). In thrombocyopenia group a higher number of patients with low Apgar score was also observed. It means that in cases of oxygen deprivation, platelet activity is inhibited, and exacerbated lung damage due to hypoxia. Low Apgar score and gestational hypertension may stimulate fetal red blood cells/megakaryocytes through acute or chronic hypoxia, affecting newborn platelet function and count. The subtending relevant mechanism needs to be further elucidated.

Recently, attention has shifted towards platelet indices such as MPV, PDW and PCT, which have become key research foci. In our study, comparing NRDS newborns with and without thrombocytopenia, those with thrombocytopenia exhibited higher PDW and MPV, and lower PCT (*p* < 0.001). PDW and MPV were inversely correlated with platelet count (rs = -0.631, -0.482, *p* all < 0.001), while PCT showed a positive correlation (rs = 0.956, *p* < 0.001). MPV is an indicator of platelet activation, reflecting platelet production and consumption dynamics [4]. PDW indicates the variability in platelet size, with larger platelets possessing more active enzymes and increased metabolism, PCT directly correlates with the overall platelet count [23]. In our study, decreased PCT, increased MPV and PDW could be associated with platelet depletion due to lung injury from NRDS and the production of younger platelets.

In this study, binary logistic regression analysis found that oxygen therapy duration (OR = 1.003, 95% CI = 1.001, 1.005), birth weight < 1500 g (OR = 3.302, 95% CI = 1.056, 10.325), gestational hypertension (OR = 2.435, 95% CI = 1.046, 5.670) and CRP levels (OR = 1.082, 95% CI = 1.029, 1.137) were indipendent risk factors for thrombocytopenia in NRDS. However, further ROC analysis revealed that oxygen therapy duration, birth weight < 1500 g, gestational hypertension and CRP levels did not exhibit strong AUC values, cut-off values, or sensitivity for predicting the occurrence of thrombocytopenia in NRDS patients. No similar studies have been found to compare our study.

### Limitations of the study

(1) This investigation is a single-center study with a relatively modest number of newborns. Future research should involve multi-center studies with larger sample sizes to validate these findings. (2) There is a scarcity of data on the relationship between platelets and NRDS for comparison, particularly regarding the evaluation of independent risk factors and ROC curves for NRDS accompanied by thrombocytopenia. (3) However, various congenital or acquired conditions that directly or indirectly contribute to thrombocytopenia should also be considered in the exclusion criteria [24–31]. Due to sample size constraints and incomplete information, this study did not examine all factors. (4) The research was conducted through clinical data analysis without foundational research, such as animal studies, thereby limiting the ability to determine specific pathogenic mechanisms. Further in-depth investigation is necessary to explore these aspects.

## Conclusions

The results of this study demonstrate that NRDS with thrombocytopenia predicts more severe disease and worse clinical outcomes. The pathogenesis of NRDS is complex and multifactorial, especially in cases associated with thrombocytopenia. Therefore, besides the deficiency of primary surfactant, we need to be vigilant about the multiple blows of various secondary factors on immature lungs. These findings are helpful for clinicians working both in I and II level birthing centers to judge the patient’s condition more accurately and to make more precise prognosis evaluation and individualized management of affected subjects [32, 33]. The exact pathogenesis of platelets dysfunction and NRDS is currently unknown and requires further study. Our findings however highlight how neonatologists should not only focus on platelet counts, but should comprehensively evaluate the patient’s conditions and risk factors to effectively and promptly treat NRDS.

## Electronic supplementary material

Below is the link to the electronic supplementary material.

Supplementary Material 1

## Data Availability

The datasets used and/or analysed during the current study are available from the first author on reasonable request.

## Abbreviations

RDS: Neonatal respiratory distress syndrome
PS: Pulmonary surfactant
PDW: Platelet distribution width
PCT: Platelet crit
MPV: Mean platelet volume
AUC: Area under curve
NICU: Neonatal intensive care unit
OR: Odds ratio
ROC: Receiver operating characteristics curve
CI: Confdence interval
CRP: C-reactive protein
WBC: White blood cell
