# Supplementary material for: Relationship between platelet count and severity of neonatal respiratory distress syndrome
Source: Ital J Pediatr. 2024 Oct 8;50:208. doi: 10.1186/s13052-024-01762-2 (PMC11462692; doi:10.1186/s13052-024-01762-2)
Supplement: Supplementary file 1 — Supplementary Material 1 [file 13052_2024_1762_MOESM1_ESM.docx]

Dear Reviewer

Thank you very much for your time viewing the response, our manuscript “Relationship between platelet count and severity of neonatal respiratory distress syndrome” (Manuscript ID: ITJP-D-24-00010) was recommended a minor revision, we have modified the manuscript already according to the referees’ kind comments, and the detailed corrections are listed below point by point. The manuscript has been resubmitted to *Italian Journal of Pediatrics*. We are looking forward to your positive response.

With kindest regards,

Yours Sincerely,

Ying Zeng

**Reply to reviewer 2**

**Abstract**

***1. The study aimed to analyze (and not aims, for consistency with the other tenses used)***

**Reply:** Thanks for reviewer’s kind suggestion. They have been revised, see red fonts in the abstract section.

After modification: This study aimed to analyze the clinical characteristics of NRDS with thrombocytopenia, explore the...

Original: This study aims to analyze the clinical characteristics of NRDS with thrombocytopenia, explore the ...

***2. CRP, explain in full***

**Reply:** Thanks for reviewer’s valuable advice. They have been revised, see red fonts in the abstract section.

After modification: …and C-reactive protein (CRP) levels were inversely associated with platelet count

Original:…and CRP were inversely associated with platelet count

***3.whose mothers had gestational hypertension***

**Reply:** Thanks for review’s kind remind. They have been revised, see red fonts in the abstract section.

After modification: … whose mothers had gestational hypertension.

Original: …whose mothers with gestational hypertension.

**Main manuscript**

**Background**

***4.*** ***investigate (to avoid the repetition explore) the diagnostic capability of variables? (please clarify variables) for reduced platelets counts.***

**Reply:** Thanks for review’s kind remind. They have been revised, see red fonts in the Background section.

After modification: … investigate the diagnostic capability of C-reactive protein (CRP), birth weight <1500 g, oxygen therapy duration, and gestational hypertension for reduced platelet counts.

Original:… explore the diagnostic capability of variables for reduced platelet counts in NRDS.

**Methods**

***5. the chest radiograph with a score 1 to 4, please amend and clarify this sentence***

***chest radiograph findings, please correct.***

**Reply:** Thanks for reviewer’s valuable remind. They have been revised, see red fonts in the Methods (Participants).

After modification: … the chest radiograph findings with a score 1 to 4.

Original:…the chest radiograph with a score 1 to 4.

***6.*** ***Add a comma after "hypoxic-ischemic encephalopathy".***

**Reply:** Thanks for reviewer’s valuable remind. They have been revised, see red fonts in the Methods (**Exclusion criteria**).

After modification: Perinatal asphyxia complications, including hypoxic-ischemic encephalopathy, acute tubular necrosis, transient myocardial ischemia.

Original: Perinatal asphyxia complications, including hypoxic-ischemic encephalopathy acute tubular necrosis, transient myocardial ischemia.

***7. thrombocytopenic purpura, please correct***

**Reply:** Thanks for reviewer’s kind remind. They have been revised, see red fonts in the Methods (**Exclusion criteria**).

After modification: …thrombocytopenic purpura.

Original:…immune hrombocytopenic purpura.

***8. accounts for 75% of cases in newborns (to avoid the repetition thrombocytopenia)***

**Reply:** Thanks for reviewer’s kind remind. They have been revised, see red fonts in the Methods (**Groups classification**).

After modification: …and such early thrombocytopenia accounts for 75% of cases in newborns admitted to NICU.

Original: …and such early thrombocytopenia accounts for 75% of thrombocytopenia in newborns admitted to NICU.

**Results**

***9. please remove "after birth" after "Apgar score", as it is redundant.***

**Reply:** Thanks for reviewer’s valuable suggestion. They have been removed (**Baseline characteristics, Results**).

After modification: …and low Apgar score occurred in more newborns.

Original: …and low Apgar score after birth occurred in more newborns.

***10.*** ***avoid the repetition "the thrombocytopenia group", use instead the patients belonging to this latter bracket, or party***

**Reply:** Thanks for reviewer’s valuable advice. They have been revised, see red fonts in the **Results** section (Clinical features).

After modification: The hospital stay in the thrombocytopenia group was 13.5 days longer than in the non-thrombocytopenia group (*p* < 0.001). The ventilation time was 18 hours longer in the thrombocytopenia group compared to the non-thrombocytopenia group (*p* < 0.05). The invasive ventilation time was 27.5 hours longer in the patients belonging to this latter bracket, though this difference was not statistically significant (*p* > 0.05). The non-invasive ventilation time was 7.5 hours longer in the patients belonging to this latter bracket (*p* < 0.05). Oxygen therapy duration was 144 hours longer in the patients belonging to this latter party (*p* < 0.001). The thrombocytopenia group had more PS administrations (*p* < 0.05).

Original: The hospital stay in the thrombocytopenia group was 13.5 days longer than in the non-thrombocytopenia group (*p* < 0.001). The ventilation time was 18 hours longer in the thrombocytopenia group compared to the non-thrombocytopenia group (*p* < 0.05). The invasive ventilation time was 27.5 hours longer in the thrombocytopenia group, though this difference was not statistically significant (*p* > 0.05). The non-invasive ventilation time was 7.5 hours longer in the thrombocytopenia group (*p* < 0.05). Oxygen therapy duration was 144 hours longer in the thrombocytopenia group (*p* < 0.001). The thrombocytopenia group had more PS administrations (*p* < 0.05).

11. ***tables 2 and 3, amend thrombocytopenia*** ***(table 3), and remove "The" before the items in which it is currently present***

**Reply:** Thanks for reviewer’s valuable advice. They have been revised. see red fonts in the **Results** section (Clinical features). **Figure 1(F)** and **Figure 2** heve also been revised.

After modification:

| **Table 2** Comparison of clinical features between the two groups | | | | |
| --- | --- | --- | --- | --- |
| **Item** | **Thrombocytopenia group (n=50)** | **Non-thrombocytopenia group (n=184)** | **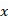^2^ /Z** | ***P-*value** |
| Hospital stay (d) | 24.5 (12.0, 43.3) | 11.0 (9.0, 18.8) | -4.570 | ＜0.001*** |
| Pulse (bpm) | 152.0 (145.0, 158.0) | 152.5 (150.0, 156.0) | -0.497 | 0.619 |
| Respiratory rate (bpm) | 62.0 (60.0, 65.0) | 62.0 (59.3, 65.0) | -0.719 | 0.472 |
| Ventilation time (h) | 88.0 (52.5, 202.3) | 70.0 (47.0, 108.5) | -2.217 | 0.027* |
| Invasive ventilation time (h) | 38.0 (0.0, 82.5) | 10.5 (0.0, 47.8) | -1.851 | 0.064 |
| Non-invasive ventilation time (h) | 54.5 (39.8, 103.8) | 47.0 (32.3, 71.0) | -1.969 | 0.049* |
| Oxygen therapy duration (h) | 301.0 (156.3, 628.3) | 157.0 (107.3, 259.0) | -4.005 | ＜0.001*** |
| PS administrations |  |  |  |  |
| 0-1 times | 39.0 (78.0%) | 165.0 (89.7%) | 4.794 | 0.029* |
| 2-3 times | 11.0 (22.0%) | 19.0 (10.3%) |  |  |
| Note: * ***P***＜0.05, *** ***P***＜0.001 | | | | |

| **Table 3** Further comparison of clinical features in thrombocytopenia group | | | | |
| --- | --- | --- | --- | --- |
| **Item** | **Mild (n=46)** | **Moderate - severe (n=4)** | **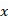^2^ /Z** | ***P-*value** |
| Hospital stay (d) | 21.5 (11.5, 35.3) | 48.5 (47.3, 60.3) | -2.469 | 0.014* |
| Pulse (bpm) | 152.0 (145.8, 158.0) | 150.0 (144.3, 155.8) | -0.681 | 0.496 |
| Respiratory rate (bpm) | 63.0 (60.0, 65.0) | 59.5 (25.8, 61.8) | -1.875 | 0.061 |
| Ventilation time (h) | 88.0 (51.3, 202.3) | 151 (69.8, 687.5) | -0.966 | 0.334 |
| Invasive ventilation time (h) | 38.0 (0.0, 82.5) | 23.0 (0.0, 389.5) | -0.056 | 0.956 |
| Non-invasive ventilation time(h) | 53.0 (38.5, 84.5) | 128 (61.8,306.0) | -1.789 | 0.074 |
| Oxygen therapy duration (h) | 279.5 (116.3, 574.5) | 800.5 (426.3, 1174.8) | -2.038 | 0.042* |
| PS administrations |  |  |  |  |
| 0-1 times | 37.0 (80.4%) | 2.0 (50.0%) | 3.588 | 0.250 |
| 2-3 times | 9.0 (19.6%) | 2.0 (50.0%) |  |  |
| Note: * ***P***＜0.05 | | | | |

Original:

| **Table 2** Comparison of clinical features between the two groups | | | | |
| --- | --- | --- | --- | --- |
| **Item** | **Thrombocytopenia group (n=50)** | **Non-thrombocytopenia group (n=184)** | **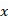^2^ /Z** | ***P-*value** |
| Hospital stay (d) | 24.5 (12.0, 43.3) | 11.0 (9.0, 18.8) | -4.570 | ＜0.001*** |
| Pulse (bpm) | 152.0 (145.0, 158.0) | 152.5 (150.0, 156.0) | -0.497 | 0.619 |
| Respiratory rate (bpm) | 62.0 (60.0, 65.0) | 62.0 (59.3, 65.0) | -0.719 | 0.472 |
| The ventilation time (h) | 88.0 (52.5, 202.3) | 70.0 (47.0, 108.5) | -2.217 | 0.027* |
| The invasive ventilation time (h) | 38.0 (0.0, 82.5) | 10.5 (0.0, 47.8) | -1.851 | 0.064 |
| The non-invasive ventilation time (h) | 54.5 (39.8, 103.8) | 47.0 (32.3, 71.0) | -1.969 | 0.049* |
| Oxygen therapy duration (h) | 301.0 (156.3, 628.3) | 157.0 (107.3, 259.0) | -4.005 | ＜0.001*** |
| PS administrations |  |  |  |  |
| 0-1 times | 39.0 (78.0%) | 165.0 (89.7%) | 4.794 | 0.029* |
| 2-3 times | 11.0 (22.0%) | 19.0 (10.3%) |  |  |
| Note: * ***P***＜0.05, *** ***P***＜0.001 | | | | |

| **Table 3** Further comparison of clinical features in hrombocytopenia group | | | | |
| --- | --- | --- | --- | --- |
| **Item** | **Mild (n=46)** | **Moderate - severe (n=4)** | **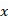^2^ /Z** | ***P-*value** |
| Hospital stay (d) | 21.5 (11.5, 35.3) | 48.5 (47.3, 60.3) | -2.469 | 0.014* |
| Pulse (bpm) | 152.0 (145.8, 158.0) | 150.0 (144.3, 155.8) | -0.681 | 0.496 |
| Respiratory rate (bpm) | 63.0 (60.0, 65.0) | 59.5 (25.8, 61.8) | -1.875 | 0.061 |
| The ventilation time (h) | 88.0 (51.3, 202.3) | 151 (69.8, 687.5) | -0.966 | 0.334 |
| The invasive ventilation time (h) | 38.0 (0.0, 82.5) | 23.0 (0.0, 389.5) | -0.056 | 0.956 |
| The non-invasive ventilation time(h) | 53.0 (38.5, 84.5) | 128 (61.8,306.0) | -1.789 | 0.074 |
| Oxygen therapy duration (h) | 279.5 (116.3, 574.5) | 800.5 (426.3, 1174.8) | -2.038 | 0.042* |
| PS administrations |  |  |  |  |
| 0-1 times | 37.0 (80.4%) | 2.0 (50.0%) | 3.588 | 0.250 |
| 2-3 times | 9.0 (19.6%) | 2.0 (50.0%) |  |  |
| Note: * ***P***＜0.05 | | | | |

.

***12.- hypersensitive C-reactive protein??, please clarify***

**Reply:** Thanks for reviewer’s kind suggestion. They have been revised and moved, see red fonts in the Background section.

After modification: CRP levels.

Original: hypersensitive C-reactive protein (CRP).

***13.-*** ***Negative correlations and positive ones must be better explained, as did in the discussion section.***

***whose mothers were affected by gestational hypertension***

**Reply:** Thanks for reviewer’s kind remind. They have been revised, see red fonts in the **Results** section (**Correlation analysis between platelet…whole study sample (n=234)**.

After modification: The study found negative correlations between platelet count and hospital stay, ventilation time, oxygen therapy duration, chest radiography score, CRP, PDW, MPV (rs = -0.326, -0.184, -0.257, -0.154, -0.323, -0.631, -0.482, *p*＜0.001, = 0.005,＜0.001, = 0.018,＜0.001,＜0.001,＜0.001, respectively). Conversely, positive correlations were observed between platelet count and Apgar scores at 1 or 5 minutes, gestational age, birth weight, and PCT (rs = 0.252, 0.280, 0.217, 0.240, 0.956, *p*＜0.001,＜0.001, = 0.001,＜0.001,＜0.001, respectively).In NRDS, the occurrence of thrombocytopenia was associated with longer ventilation time, oxygen therapy duration and hospital stay. Those with thrombocytopenia exhibited higher PDW, MPV, CRP levels and chest radiography score, and lower PCT, birth weight, 1-minute and 5-minute Apgar scores, and younger in gestational age (Figure 1). Point-biserail correlation showed that thrombocytopenia was more likely to occur in newborns whose mothers were affected by gestational hypertension (correlation coefficient was -0.155, *p* =0.018).

Original: The study found negative correlations between platelet count and hospital stay, ventilation time, oxygen therapy duration, chest radiography score, CRP, PDW, MPV (rs = -0.326, -0.184, -0.257, -0.154, -0.323, -0.631, -0.482, *p*＜0.001, = 0.005,＜0.001, = 0.018,＜0.001,＜0.001,＜0.001, respectively). Conversely, positive correlations were observed between platelet count and Apgar scores at 1 or 5 minutes, gestational age, birth weight, and PCT (rs = 0.252, 0.280, 0.217, 0.240, 0.956, *p*＜0.001,＜0.001, = 0.001,＜0.001,＜0.001, respectively) (Figure 1). Point-biserail correlation showed that thrombocytopenia was more likely to occur in newborns whose mothers were affected by gestational hypertension (correlation coefficient was -0.155, *p* =0.018).

**Discussion**

***14.change "more newborns with chest radiography scores of 3-4 point" with "worst chest radiography severity scores (3-4)"***

**Reply:** Thanks for reviewer’s kind suggestion. They have been revised, see red fonts in the Discussion section.

After modification: …worst chest radiography severity scores (3-4) and more PS administrations.

Original: …more newborns with chest radiography scores of 3-4 points and PS administrations.

***15.amend "halina"***

**Reply:** Thanks for reviewer’s valuable remind. We reviewed the paper more seriously and corrected them, see red fonts in the Discussion.

After modification: …lead to fibrin deposition and hyaline membrane formation in severe NRDS.

Original: …lead to fibrin deposition and hyalina membrane formation in severe NRDS.

***16.-*** ***write Studies in lowercase***

**Reply:** Thanks for reviewer’s valuable remind. The relevant word has been corrected, see red fonts in the Discussion section.

After modification: In addition, studies have found that beyond primary surfactant deficiency...

Original: In addition, Studies have found that beyond primary surfactant deficiency...

***17.-*** ***change "it's" with "it is"***

**Reply:** Thanks for reviewer’s valuable suggestion. We reviewed the paper more seriously and corrected them, see red fonts in the Discussion.

After modification: it is widely accepted that hypertension...

Original: it's widely accepted that hypertension...

***18.-*** ***write low Apgar score in uppercase***

**Reply:** Thanks for reviewer’s valuable suggestion. The relevant word has been corrected, see red fonts in the Discussion.

After modification: …damage due to hypoxia. Low Apgar score and gestational hypertension may…

Original: …damage due to hypoxia. low Apgar score and gestational hypertension may…

**Limitations of the study**

***19. Limitations of the study (in uppercase).***

**Reply:** Thanks for reviewer’s valuable suggestion. The relevant word has been corrected, see red fonts.

After modification: **Limitations of the study**

Original: **limitations of the study.**

***20.- point 3 appears detached from the context, please improve this issue***

**Reply:** Thanks for reviewer’s valuable suggestion. The relevant word has been corrected, see red fonts in the **Limitations of the study**.

After modification: 3. However, various congenital or acquired conditions that directly or indirectly contribute to thrombocytopenia should also be considered in the exclusion criteria. Due to sample size constraints and incomplete information, this study did not examine all factors.

Original: 3. However, they also should consider and include various congenital or acquired conditions associated with or contributing directly or indirectly to thrombocytopenia. Due to sample size constraints and incomplete information, this study did not examine these factors.

**Conclusions**

***21.- The exact pathogenesis of platelets*** ***dysfunction and NRDS is currently unknown...(add dysfunction)***

**Reply:** Thanks for reviewer’s valuable suggestion. The relevant word has been corrected, see red fonts in the **Conclusions**.

After modification: The exact pathogenesis of platelets dysfunction and NRDS is currently unknown and requires further study.

Original: The exact pathogenesis of platelets and NRDS is currently unknown and requires further study.

***22.- evaluate the patient's conditions*** ***and risk factors to effectively and promptly treat NRDS.***

**Reply:** Thanks for reviewer’s valuable suggestion. The relevant word has been corrected, see red fonts in the **Conclusions**.

After modification: but should comprehensively evaluate the patient's conditions and risk factors to effectively and promptly treat NRDS.

Original: but should comprehensively evaluate the patient's condition to effectively and promptly treat NRDS.
